# Supplementary figures and images for: Comparison of transgenic and adenovirus hACE2 mouse models for SARS-CoV-2 infection
Source: Emerg Microbes Infect. 2020 Nov 6;9(1):2433–45. doi: 10.1080/22221751.2020.1838955 (PMC7655046; doi:10.1080/22221751.2020.1838955)

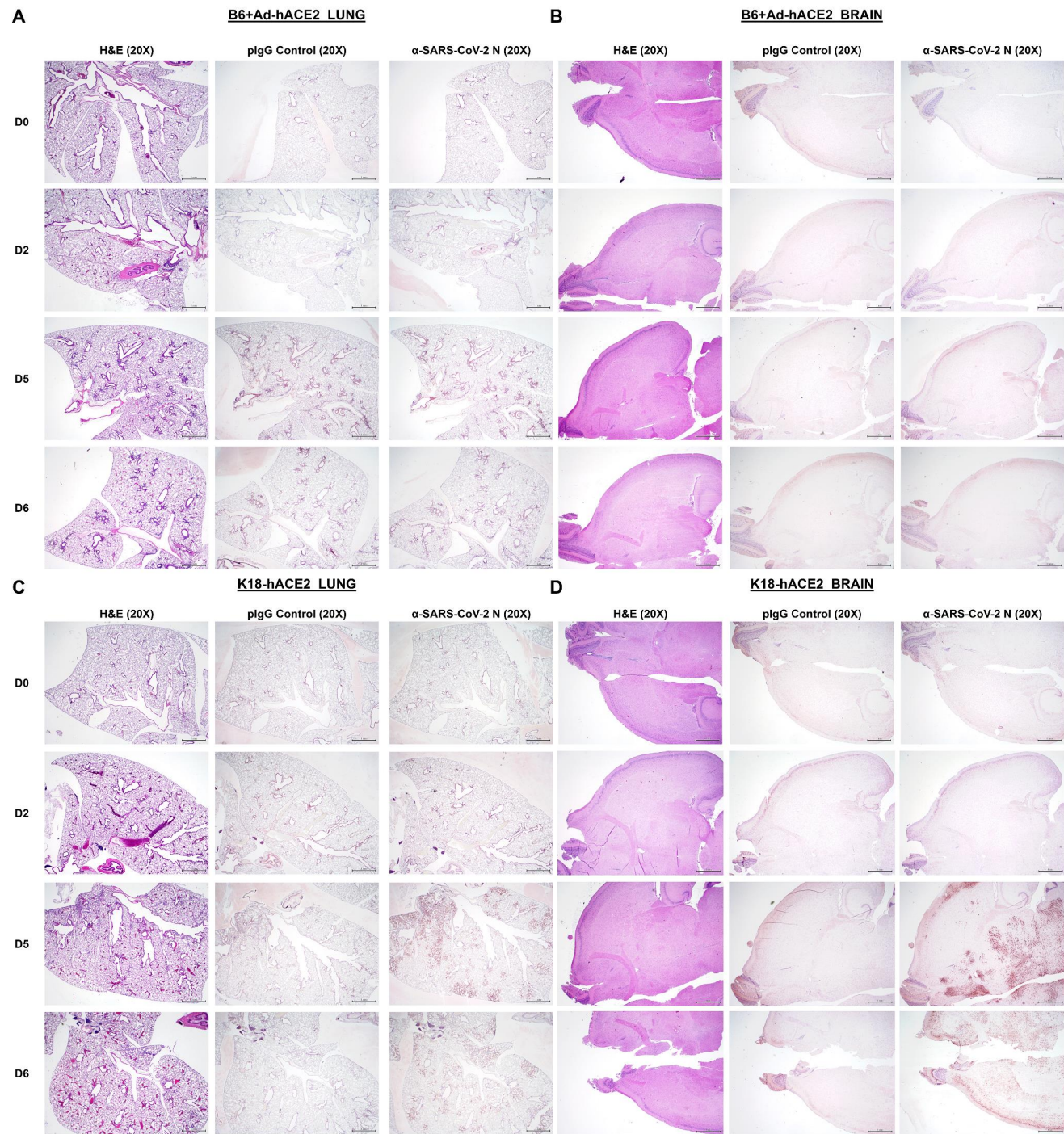

Supplement: Supplemental Material [file TEMI_A_1838955_SM3933.pdf]

# Composite Pathology Score (Lung H&E)

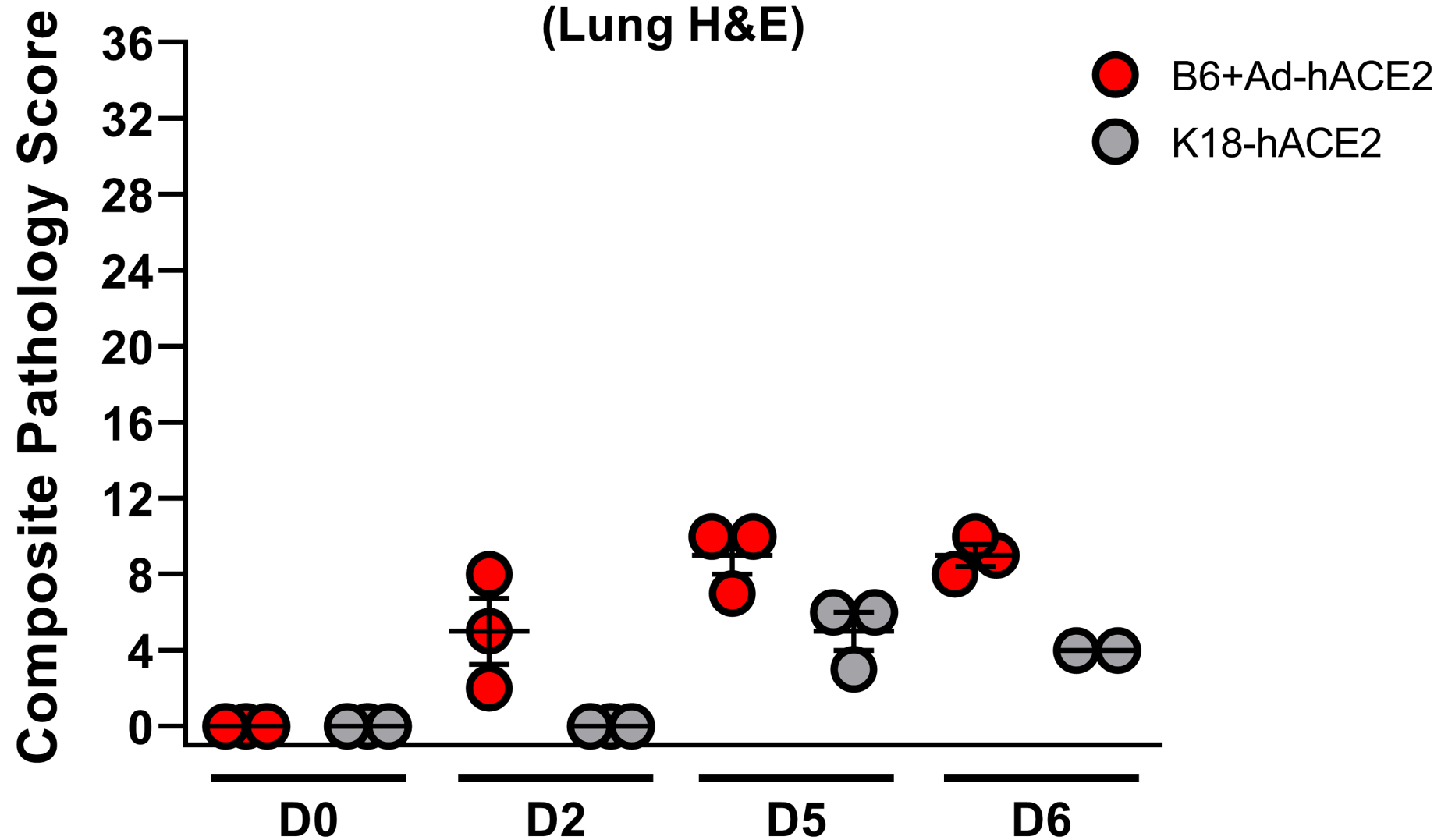

Supplement: Supplemental Material [file TEMI_A_1838955_SM3780.pdf]
